# Rethinking the Role of Formal Physical Therapy in Glenohumeral Osteoarthritis: A Nationwide Study Comprising More than Two Million Patients in the United States

**DOI:** 10.5435/JAAOSGlobal-D-24-00225

**Published:** 2024-11-19

**Authors:** Amir Human Hoveidaei, Amirhossein Ghaseminejad-Raeini, Fatemeh Kanaani Nejad, Fatemeh Moosaie, Sara Mohammadi, Mohammad Saeid Khonji, Basilia Onyinyechukwu Nwankwo, Mohit N. Gilotra, Janet D. Conway

**Affiliations:** From the International Center for Limb Lengthening, Rubin Institute for Advanced Orthopedics, Sinai Hospital of Baltimore, Baltimore, ML (Dr. Hoveidaei, Dr. Nwankwo, and Dr. Conway); School of Medicine, Tehran University of Medical Sciences, Tehran, Iran (Dr. Ghaseminejad-Raeini, Dr. Moosaie and Dr. Mohammadi); Anesthesiology and Critical Care Research Center, Shiraz University of Medical Sciences, Shiraz, Iran (Dr. Kanaani Nejad); Bone and Joint Reconstruction Research Center, Iran University of Medical Sciences, Tehran, Iran (Dr. Saeid Khonji); Department of Orthopaedic Surgery and Rehabilitation, Howard University Hospital, Washington, DC (Dr. Nwankwo); the Department of Orthopaedics, University of Maryland School of Medicine, Baltimore, BL (Dr. Gilotra). Correspondence to Dr. Conway: jconway@lifebridgehealth.org

## Abstract

**Introduction:**

This study aims to determine whether incorporating physical therapy into a nonsurgical approach can effectively manage the course of glenohumeral osteoarthritis (OA) and potentially prevent patients from requiring total shoulder arthroplasty (TSA).

**Methods:**

This retrospective cohort consisted of patients diagnosed with glenohumeral OA between 2010 and 2021 using ICD-9, ICD-10, and current procedural terminology codes in the PearlDiver database. Two- and five-year TSA rates were compared between patients who had physical therapy within 1 year after glenohumeral OA diagnosis and patients who did not.

**Results:**

The study consisted of 2,710,463 patients with glenohumeral OA. After propensity score matching, among patients with corticosteroid injection, patients who received physical therapy had significantly higher 2- and 5-year TSA rates compared with those without physical therapy (2-year TSA: 0.60% vs. 0.35%, OR [95% CI]: 1.74 [1.50, 2.01] and 5-year TSA: 1.13% vs. 0.65%, OR [95% CI]: 1.74 [1.56, 1.93]). A similar association was also significant among patients without corticosteroid injection (0.35% vs. 0.16%, OR [95% CI]: 2.17 [2.03, 2.21] and 0.78% vs. 0.37%, OR [95% CI]: 2.11 [2.01, 2.21]).

**Conclusion:**

Individuals who had physical therapy as a part of their nonsurgical treatment did not have any decrease in the probability of requiring TSA.

Glenohumeral osteoarthritis (OA) is a degenerative condition characterized by the gradual deterioration of articular cartilage, leading to the erosion of bone, persistent pain, and impaired physical function.^1^ Approximately up to 21% of radiologic observations among middle-aged and elderly populations include features of joint damage associated with glenohumeral OA.^2^ Considering the prevalence of glenohumeral OA, its substantial effects on patients' quality of life, and the considerable allocation of healthcare resources toward different treatment modalities,^3^ it is evident that there is a pressing need to establish an optimal treatment strategy for individuals diagnosed with glenohumeral OA.

Currently, there is no cure for patients diagnosed with OA. However, surgical and nonsurgical approaches are employed to manage pain and effectively preserve physical function.^4^ Nonsurgical plans encompass a range of approaches aimed at the primary management of glenohumeral OA. These include oral anti-inflammatory drugs, intra-articular injection of corticosteroids, and physical therapy, such as manual physical therapy, exercises, and progressive functional activities, which are mainly incorporated into a multidisciplinary nonsurgical treatment plan.^5,6^ Surgical strategies, such as total shoulder arthroplasty (TSA), are employed for those who have failed the nonsurgical treatments.^6^ According to recent studies, incorporating physical therapy into a nonsurgical treatment regimen can reduce discomfort and protect the glenohumeral joint.^7^ Others claim that physical therapy, regardless of the severity of glenohumeral OA, may not improve patients' clinical condition.^8^

The clinical practice guideline for the management of glenohumeral joint OA released by the American Academy of Orthopaedic Surgeons in 2020, while pointing to the absence of reliable evidence, includes a consensus recommendation advocating for preoperative physical therapy for patients diagnosed with glenohumeral OA.^9^ Considering that treatment plans are designed based on clinical practice guidelines, to reduce ineffective practice variation and optimize patients' care, solid evidence is needed to appropriately advise physicians about the efficacy of recommendations.^10^

This nationwide study conducted in the United States aims to compare the rate of 2- and 5-year TSA among patients diagnosed with glenohumeral OA who had physical therapy in their nonsurgical treatment plan with those who did not. This would determine whether incorporating physical therapy into a nonsurgical approach could effectively prevent or delay patients from undergoing TSA surgery.

## Methods

### Data Source

Patients' records were queried from PearlDiver (PearlDiver Inc, Fort Wayne, IN, USA), a commercially available administrative claims database, using International Classification of Diseases ninth Revision (ICD-9) and 10th Revision (ICD-10), as well as procedures or current procedural terminology (CPT) codes. This study used the M-161 Ortho data set, a subset of the Mariner database that contains the medical records of more than 100 million patients. Patient information from all sources was deidentified and adhered to the Health Insurance Portability and Accountability Act regulations. The study was exempt from institutional review board approval.

### Study Population

**Figure 1:**
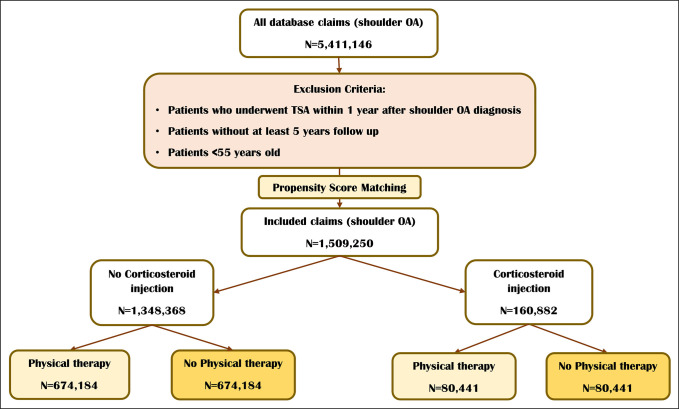
Flowchart showing patient selection.

The M-161 Ortho data set was employed for the data extraction involving patients with glenohumeral OA diagnosis from 2010 to 2021. A retrospective cohort design was used to compare patients who had physical therapy within 1 year after glenohumeral OA diagnosis vs. those who did not receive physical therapy among patients with and without corticosteroid injection in 1 year after diagnosis. Inclusion criteria were a diagnosis of glenohumeral OA made by board-certified physicians and defined by ICD diagnosis codes in the database. Patients who underwent TSA within a single year after glenohumeral OA diagnosis, aged <55 years old, and without at least 5 years of follow-up were excluded. Patients who had shoulder corticosteroid injections and those who received physical therapy were identified using both ICD and CPT codes. Because no specific code was found for corticosteroid injection, we used a combination of ICD and CPT codes to identify them. Initially, codes for large joint injections were used, then codes for shoulder pain a year prior were queried. Eventually, the CPT codes related to corticosteroids used for intra-articular injection were queried to filter out patients with corticosteroid injection. ICD and CPT codes that defined the study groups are provided in Supplementary Table 1, http://links.lww.com/JG9/A374 in more detail. The patient selection flow chart is provided in Figure 1.

### Variables and Outcomes

Each cohort was queried for variables, including basic demographic information, clinical characteristics, and comorbidities, that is, age, sex, Charlson Comorbidity Index (CCI), tobacco use, obesity, and diabetes mellitus (Dm) using ICD diagnosis codes.

The outcomes of the study were incidences of 2- and 5-year TSA, defined as having TSA in 2 and 5 years after glenohumeral OA diagnosis, respectively, and were queried for the two patient cohorts using the codes CPT-23472, ICD-9-P-8180, ICD-9-P-8188, ICD-10-P-0RRJ00Z, ICD-10-P-0RRJ0JZ, ICD-10-P-0RRJ0J6, ICD-10-P-0RRK00Z, ICD-10-P-0RRK0J6, ICD-10-P-0RRK0JZ, ICD-10-P-0RRJ07Z, ICD-10-P-0RRJ0KZ, ICD-10-P-0RRK07Z, and ICD-10-P-0RRK0KZ.

### Statistical Analysis

R statistical software (R Project for Statistical Computin) was employed for the data analysis, integrated within PearlDiver with an α level set to 0.05. For the univariate analysis, to determine the association between demographics and comorbidities in the patient groups, the *t*-test was used for quantitative variables and the Chi-square for qualitative variables. Continuous variables were presented as mean ± SD, and categorical variables were presented as frequency (%). For the purpose of adjustment for the confounding variables, propensity score matching was employed. Logistic regression was conducted for comparison of 2- and 5-year TSA between patients having and not having physical therapy and among two subgroups of with and without corticosteroid injection. Matching was done for age, sex, CCI, obesity, tobacco use, and diabetes. It is important to take into account the effect that age, sex, CCI, obesity, tobacco use, and diabetes can have on the 2- and 5-year incidence of TSA. The decision to control for the aforementioned variables in the statistical analysis was because each variable has substantial potential for exerting confounding effects given the associated risks that each has on the incidence of TSA in patients with the diagnosis of glenohumeral OA. Patients with DM and CCI above three have a higher prevalence of unsuccessful physical therapy compared with those without DM.^11^ Studies show poor physical therapy outcomes in patients who smoke, through its hypoxic effects and impaired perfusion to organ systems.^12-14^ Patients with obesity have shown less significant functional improvement during rehabilitation.^15^

## Results

### Patient Selection and Baseline Characteristics

The study consisted of 2,710,463 patients with a diagnosis of glenohumeral OA, 182,998 with corticosteroid injection within 1 year after diagnosis, and 2,527,465 without corticosteroid injection. Among patients with corticosteroid injection, 80,441 underwent physical therapy within 1 year after glenohumeral OA diagnosis, and 102,557 received no physical therapy. Among patients without corticosteroid injection, 674,184 received physical therapy within 1 year after diagnosis, and 1,853,281 received no physical therapy. The mean age was 67.33 and 68.93 years among patients with corticosteroid injection with and without physical therapy (*P* < 0.001) and 67.10 and 68.42 years among patients without corticosteroid injection with and without physical therapy, respectively (*P* < 0.001). All study groups were predominantly women (*P* < 0.001). The CCI score was slightly higher in patients who received physical therapy compared with those did not receive physical therapy among patients who had corticosteroid injections (mean: 2.05 vs. 2.00, *P* < 0.001). Conversely, it was slightly higher among patients without physical therapy compared with those who received physical therapy among patients without corticosteroid injection (mean: 2.01 vs. 1.96, *P* < 0.001). Obesity was more prevalent among patients with physical therapy compared with those without (47.9% vs. 39.1% and *P* < 0.001, among patients with corticosteroid injection, 42.5% vs. 35.3% and *P* < 0.001, among patients without corticosteroid injection). Diabetes (55.0% vs. 51.7%, *P* < 0.001, among patients with corticosteroid injection, 40.7% vs. 37.7% and *P* < 0.001, among patients without corticosteroid injection) and tobacco use (45.0% vs. 41.0%, *P* < 0.001 among patients with corticosteroid injection, 52.3% vs. 50.6% and *P* < 0.001, among patients without corticosteroid injection) also had a higher prevalence among patients with physical therapy compared with those who did not receive physical therapy. Table 1 presents the demographic characteristics of the participants in more detail.

**Table 1:** Demographic Variables and Comorbidities Distribution in Patients With Glenohumeral Osteoarthritis

	Corticosteroid Injection (N = 182,998)			No Corticosteroid Injection (N = 2,527,465)		
	Physical Therapy (N = 80,441)	No Physical Therapy (N = 102,557)	*P*	Physical Therapy (N = 674,184)	No Physical Therapy (N = 1,853,281)	*P*
Age, year (mean ± SD)	67.33 ± 7.40	68.93 ± 7.26	<0.001	67.10 ± 7.26	68.42 ± 7.27	<0.001
Female	47,022 (58.5%)	58,232 (56.8%)	<0.001	374,684 (55.6%)	102,4077 (55.3%)	<0.001
Male	33,419 (41.5%)	44,324 (43.2%)		299,498 (44.4%)	829,194 (44.7%)	
CCI score (mean ± SD)	2.05 ± 2.54	2.00 ± 2.50	<0.001	1.96 ± 2.57	2.01 ± 2.62	<0.001
Obesity, N (%)	38,530 (47.9%)	401,44 (39.1%)	<0.001	286,628 (42.5%)	654,903 (35.3%)	<0.001
Diabetes, N (%)	44,197 (55.0%)	53,073 (51.7%)	<0.001	274,374 (40.7%)	697,954 (37.7%)	<0.001
Tobacco use N (%)	36,169 (45.0%)	42,041 (41.0%)	<0.001	352,881 (52.3%)	937,354 (50.6%)	<0.001

N = number, SD = standard deviation, CCI = Charlson Comorbidity Index

### Two-Year Total Shoulder Arthroplasty

After matching, the association between receiving physical therapy and 2- and 5-year incidence of TSA adjusting for potential confounding variables (ie, age, sex, CCI score, obesity, tobacco use, and diabetes) was assessed. The results showed that OA cases who received corticosteroid injection and physical therapy had significantly higher 2-year incident TSA compared with those without physical therapy (2-year TSA: 0.60% vs. 0.35%, OR (95% CI): 1.74 (1.50, 2.01), *P* < 0.001). Patients without corticosteroid injection with physical therapy had a higher incidence of 2-year TSA compared with those who did not receive physical therapy (2-year TSA: 0.35% vs. 0.16%, OR (95% CI): 2.17 (2.03, 2.34), *P* < 0.001; Table 2).

**Table 2:** The Association Between Physical Therapy and Incidence of 2- and 5-Year Total Shoulder Arthroplasty Among Glenohumeral Patients With and Without Corticosteroid Injection Following Propensity Score Matching in Each Group

	Corticosteroid Injection				No Corticosteroid Injection			
	Physical Therapy	No Physical Therapy	Odds Ratio^a^	*P*	Physical Therapy (N = 674,184)	No Physical Therapy (N = 674,184)	Odds Ratio^a^	*P*
2-year TSA	485 (0.60%)	279 (0.35%)	1.74 (1.50, 2.01)	<0.001	2,350 (0.35%)	1,081 (0.16%)	2.17 (2.03, 2.34)	<0.001
5-year TSA	905 (1.13%)	524 (0.65%)	1.74 (1.56, 1.93)	<0.001	5,262 (0.78%)	2,497 (0.37%)	2.11 (2.01, 2.21)	<0.001

TSA = total shoulder arthroplasty

^a^The odds ratio was adjusted for age, sex, Charlson comorbidity index, obesity, tobacco use and diabetes.

Five-year total shoulder arthroplasty.

Among the patients with corticosteroid injection, a significantly higher 5-year incidence of TSA was found in patients who received physical therapy within 1 year after diagnosis, compared with the control group (5-year TSA: 1.13% vs. 0.65%, OR (95% CI): 1.74 (1.56, 1.93), *P* < 0.001). Patients without corticosteroid injections who received physical therapy revealed a higher incidence of 5-year TSA compared with those without physical therapy (5-year TSA: 0.78% vs. 0.37%, OR (95% CI): 2.11 (2.01, 2.21), *P* < 0.001; Table 2).

## Discussion

The findings of our research on the rate of TSA in 2 and 5 years following physical therapy in patients diagnosed with glenohumeral OA suggested that individuals who did physical therapy as a part of their nonsurgical treatment had a twofold higher likelihood of requiring TSA if they received corticosteroid injection and were 2.1 times more likely to undergo TSA if did not receive corticosteroid injection compared with those who did not do physical therapy (*P* < 0.001).

There is no sufficient evidence supporting the efficacy of physical therapy in delaying or preventing TSA in glenohumeral OA. Previous investigations have examined the impact of physical therapy on symptoms in individuals diagnosed with knee and hip OA. There have been documented accounts of enhanced power,^16^ physical function,^17,18^ and pain relief^19^ among patients who had exercise and physical therapy compared with those who did not. A meta-analysis conducted on 60 trials indicated that sufficient evidence had accumulated to show the significant benefit of exercise in the attenuation of symptoms among patients with osteoarthritis.^20^

The American Academy of Orthopaedic Surgeons issued the clinical practice guideline for the management of glenohumeral OA in March 2020. This guideline has put forth a limited recommendation based on quality, quantity, and the trade-offs between the benefits and harms of preoperative physical therapy. It stated that “in the absence of reliable evidence, it is the opinion of the workgroup that physical therapy may benefit select patients with glenohumeral joint osteoarthritis.” This recommendation about including preoperative physical therapy in the treatment plan of glenohumeral OA is also endorsed by the American Shoulder and Elbow Surgeons and the American Society of Shoulder and Elbow Therapists.^9,21^

The United Kingdom's National Institute for Health and Care Excellence created an evidence review for preoperative rehabilitation, published as a research recommendation in the National Institute for Health and Care Excellence guideline in June 2020. It is claimed that “no evidence was found for people who were scheduled for shoulder replacement surgery.” Although one committee member acknowledged that patients with shoulder OA could benefit from preoperative physical therapy, the committee as a whole did not reach a consensus to recommend preoperative rehabilitation for persons having TSA.^22^

In June 2023, the American Physical Therapy Association published the latest clinical practice guideline for the management of glenohumeral joint OA. This association evaluated the effect of preoperative physical therapy based on the joint's passive and active range of motion, pain, power, anthropometrics, and glenohumeral joint's specific mechanics to assess the efficacy of treatment and patient response to care. In this guideline, while addressing minimal/conflicting evidence and a lack of validating studies, it is stated that preoperative physical therapy may benefit postoperative outcomes in patients with glenohumeral OA who are undergoing TSA based on expert opinions. According to the best practice recommendation in this guideline, at least 6 weeks of preoperative physical therapy may result in pain relief, increased physical activity, and improved surgical success expectations.^23^

The reliability of formulating guidelines and making decisions regarding preoperative physical therapy for glenohumeral joint OA based on studies conducted on other joints seems uncertain, as the glenohumeral joint exhibits fundamental distinctions, such as structure, range of motion, and instability. when compared with other joints.^24,25^ According to our study, individuals with glenohumeral OA who received physical therapy as part of their primary treatment were more likely to undergo TSA within 2 and 5 years.

The estimated total national medical expenses attributed to arthritis in 2013 were $139.8 billion annually. Across expenditure categories, ambulatory care expenses, which include nonsurgical management, such as physical therapy, accounted for nearly half of arthritis-related expenditures.^26^ There is limited OA-specific cost data, and most cost estimates reflect the total cost of all forms of arthritis. However, understanding that OA is the most prevalent form of arthritis, with a growing prevalence, will clarify OA's treatment significant portion of the healthcare system costs.^27^ According to a research investigation conducted on the economic implications of glenohumeral OA in the United States, it has been documented that approximately 20% of individuals diagnosed with this condition received physical therapy during the year before TSA. The average reimbursements for these patients, covered by Medicare Advantage and Commercial insurance beneficiaries, ranged from $372 to $473 per patient.^28^

Consensus statements from reputable organizations, such as the American Academy of Orthopaedic Surgeons, the American Shoulder and Elbow Surgeons, the American Society of Shoulder and Elbow Therapists, and the American Physical Therapy Association, suggest that preoperative physical therapy may offer potential benefits in managing glenohumeral osteoarthritis. This is expected to result in increased allocation of healthcare resources. Based on our research findings, the inclusion of preoperative physical therapy not only leads to higher expenditures associated with nonsurgical treatment but also contributes to an increased likelihood of patients undergoing TSA. Consequently, this necessitates a greater allocation of resources toward both surgical and nonsurgical management strategies.

The increased rate of TSA in those who received physical therapy as part of their nonsurgical treatment plan raises questions about the efficacy of physical therapy in hindering the advancement of OA or saving patients from undergoing TSA. More studies should be performed to clarify the main explanation of this study. Various strategies can be employed in the physical therapy management of patients who have been diagnosed with glenohumeral OA. Physical modalities, such as therapeutic heat and cold, hydrotherapy, ultrasonography, and electricity, are extensively employed for pain relief.^29^ However, the physical treatment plan may also include various exercises aimed at enhancing muscle strength, stabilizing the shoulder girdle, improving range of motion, and engaging in weight-bearing activities,^30^ which are tailored to individual cases, taking into account the patient's specific requirements, clinical condition, and physical capabilities.^31^

There are inherent limitations when drawing conclusions from national databases. A common limitation is their dependence on ICD-9, -10, and/or CPT coding to identify diagnoses, comorbidities, procedures, and outcomes. Although these codes were not originally developed to serve research purposes.^32^ In addition, these codes are based on insurance claims or hospital records derived from nonmedical individuals. The annual update of ICD-9 and CPT coding and different interpretations of medical records may lead to coding variations in the database. Moreover, this study was conducted retrospectively to compare the frequency of TSA between patients who underwent preoperative physical therapy and those who did not. The evaluation of preoperative patient conditions and postoperative outcomes, such as severity of OA, pain, physical activity, strength, and quality of life, was not the objective of this study. Hence, the results of this study offer a narrow perspective on the application of physical therapy in individuals diagnosed with glenohumeral OA, highlighting the necessity for additional research to establish general statements regarding the costs and benefits of preoperative physical therapy for glenohumeral OA.

## Conclusion

The results of our study suggest that the incorporation of preoperative physical therapy into the treatment strategy of glenohumeral OA is not associated with a decreased probability of requiring TSA at the 2 and 5-year periods. As a result, orthopaedic surgeons and physical therapists should take greater caution when advising preoperative physical therapy for glenohumeral OA management.

## Supplementary Material
