# Supplementary material for: Rethinking the Role of Formal Physical Therapy in Glenohumeral Osteoarthritis: A Nationwide Study Comprising More than Two Million Patients in the United States
Source: J Am Acad Orthop Surg Glob Res Rev. 2024 Nov 19;8(11):e24.00225. doi: 10.5435/JAAOSGlobal-D-24-00225 (PMC11578197; doi:10.5435/JAAOSGlobal-D-24-00225)
Supplement: Supplementary file 1 [file jagrr-8-e24.00225-s001.docx]

| **Table S1.** Current Procedural Terminology (CPT) and International Classification of Diseases (9th and 10th version) codes used to run the analyses | |
| --- | --- |
| **Procedure or Diagnosis** | **Codes** |
| Primary Shoulder Osteoarthritis | ICD-9-D-71511, ICD-9-D-71591, ICD-10-D-M19011, ICD-10-D-M19012, ICD-10-D-M19019 |
| Shoulder Pain | ICD-9-D-71941, ICD-10-D-M25511, ICD-10-D-M25512, ICD-10-D-M25519 |
| Large joint injection | CPT-20610 |
| Intraarticular Corticosteroid | CPT-J3300, CPT-J3301, CPT-J1020, CPT-J1030, CPT-J1040, CPT-JO702, CPT-JO704, CPT-J1100, CPT-J3302, CPT-J3303, CPT-J1094 |
| Physical Therapy | CPT-29240, CPT-97001, CPT-97002, CPT-97003, CPT-97004, CPT-97010, CPT-97012, CPT-97014, CPT-97016, CPT-97018, CPT-97022, CPT-97026, CPT-97032, CPT-97033, CPT-97035, CPT-97110, CPT-97112, CPT-97113, CPT-97116, CPT-97124, CPT-97140, CPT-97150, CPT-97530, CPT-97535, ICD-9-P-9320, ICD-9-P-9321, ICD-9-P-9325, ICD-9-P-9326, ICD-9-P-9327, ICD-9-P-9329, ICD-10-P-6A550Z1, ICD-10-P-6A551Z1, ICD-10-P-6A552Z1, ICD-10-P-6A553Z1, ICD-10-P-6A554Z1, ICD-10-P-6A555Z1 |
| Total Shoulder Arthroplasty | CPT-23472, ICD-9-P-8180, ICD-9-P-8188, ICD-10-P-0RRJ00Z, ICD-10-P-0RRJ0JZ, ICD-10-P-0RRJ0J6, ICD-10-P-0RRK00Z, ICD-10-P-0RRK0J6, ICD-10-P-0RRK0JZ, ICD-10-P-0RRJ07Z, ICD-10-P-0RRJ0KZ, ICD-10-P-0RRK07Z, ICD-10-P-0RRK0KZ |
